# Comparative study of angiographic changes in diabetic and non-diabetic patients with peripheral arterial disease

**DOI:** 10.1590/1677-5449.202000532

**Published:** 2023-02-10

**Authors:** Giovanni Ortale Trainotti, Jamil Victor Mariúba, Matheus Bertanha, Marcone Lima Sobreira, Ricardo de Alvarenga Yoshida, Rodrigo Gibin Jaldin, Paula Angeleli Bueno de Camargo, Winston Bonetti Yoshida

**Affiliations:** 1 Universidade Estadual Paulista - UNESP, Faculdade de Medicina de Botucatu, Hospital das Clínicas, Botucatu, SP, Brasil.

**Keywords:** digital angiography, peripheral vascular diseases, diabetes

## Abstract

**Background:**

Diabetics are at 5-15 times greater risk of developing peripheral arterial disease (PAD) and few studies have compared risk factors and distribution and severity of arterial changes in diabetics compared with non-diabetics.

**Objectives:**

To compare angiographic changes between diabetic and non-diabetic patients with advanced PAD and correlate them with risk factors.

**Methods:**

A retrospective cross-sectional study was conducted of consecutive patients undergoing lower limb arteriography for PAD (Rutherford 3-6) using TASC II and Bollinger et al. angiographic scores. Exclusion criteria were upper limb angiographies, unclear images, incomplete laboratory test results, and previous arterial surgeries. Statistical analyses included chi-square tests, Fisher's test for discrete data, and Student’s *t* test for continuous data (significance level: p < 0.05).

**Results:**

We studied 153 patients with a mean age of 67 years, 50.9% female and 58.2% diabetics. A total of 91 patients (59%) had trophic lesions (Rutherford 5 or 6) and 62 (41%) had resting pain or limiting claudication (Rutherford 3 and 4). Among diabetics, 81.7% were hypertensive, 29.4% had never smoked, and 14% had a history of acute myocardial infarction. According to the Bollinger et al. score, infra-popliteal arteries were more affected in diabetics, especially the anterior tibial artery (p = 0.005), while the superficial femoral artery was more affected in non-diabetics (p = 0.008). According to TASC II, the most severe angiographic changes in the femoral-popliteal segment occurred in non-diabetic patients (p = 0.019).

**Conclusions:**

The most frequently affected sectors were the infra-popliteal sectors in diabetics and the femoral sector in non-diabetics.

## INTRODUCTION

Atherosclerosis is the principal cause of cardiovascular diseases,^1^^,^ 2 a group of conditions that includes coronary artery disease, peripheral arterial disease (PAD), and cerebrovascular disease.^3^ If all of its manifestations were considered a single pathological entity, atherosclerosis would be considered the number one cause of death worldwide, estimated at 1/3 of all deaths both globally^4^ and in Brazil.^5^

Systemic arterial hypertension (SAH), diabetes mellitus (DM), and smoking significantly aggravate degeneration of the artery wall^1^^-^ 3 ^,^ 6 and control of these conditions has a powerful influence on patient prognosis.^7^^-^ 9

Peripheral arterial disease primarily affects elderly patients with risk factors for atherosclerosis^6^^,^ 9 and manifests insidiously with progressive intermittent claudication.^7^ Development of pain at rest, ischemic ulcers, and/or gangrene characterizes critical limb ischemia (CLI), which occurs in around 5-10% of cases.^9^ The best treatment option in these cases tends to be revascularization of the limb involved, except in patients with severe comorbidities or very limited prognosis of successful revascularization, for whom amputation is the most appropriate treatment.^10^^-^ 15

More than half of PAD patients have an ankle-brachial index (ABI) of < 0.9 and are asymptomatic. Of every 100 patients with intermittent claudication, 25% will progress to worse claudication, 5-10% will undergo revascularization surgery, 2-5% will undergo an amputation, and around 30% will die.^9^^,^ 13 ^,^ 16 Diabetics have around 5 to 15 times greater risk of developing PAD than people who are not diabetic.^12^^,^ 14 ^,^ 17 In patients with CLI, an arterial imaging study is recommended to best characterize the lesion and for planning surgery. Arteriography is the supplementary diagnostic examination of choice for investigation of peripheral arterial circulation.^18^^-^ 21

The few studies that have compared arterial injuries in patients with PAD and DM have shown that among diabetics arterial injuries tend to be concentrated in smaller caliber arteries (infrapopliteal).^14^^,^ 18 ^,^ 19 ^,^ 22 ^-^ 24 However, none of these studies used angiographic scores to classify lesions and correlate them with risk factors and demographic data.

Our objective, therefore, was to quantify and compare angiographic changes in diabetic and non-diabetic patients with advanced PAD using scores and correlate them with other risk factors.

## MATERIALS AND METHODS

A retrospective, cross-sectional, observational study was conducted of a consecutive series of cases, analyzing arteriographies conducted from 2012 to December of 2016 at a single center, comparing diabetic and non-diabetic patients. The study was approved by the local Ethics Committee (decision number 1.578.037).

Consecutive patients with advanced PAD (Rutherford 3 to 6) with and without diabetes were enrolled. Briefly, the Rutherford classification^25^ consists of the following categories: 0 = asymptomatic; 1 = mild claudication; 2 = moderate claudication; 3 = severe claudication; 4 = pain at rest; 5 = small trophic lesion; 6 = extensive necrosis. Categories from 3 to 6 were arbitrarily defined as advanced for the present study. Exclusion criteria included angiographs of upper limbs, patients who had undergone surgical revascularization interventions, incomplete laboratory test results, and unclear angiographs.

Demographic data were collected from the hospital’s MV electronic patient record system, including age, ethnicity self-declared on the patient record, comorbidities, and laboratory test results (urea, creatinine, glycemia, and lipid profile). Clinical complaints reported by the patients and confirmed by the physicians were as follows: pain at rest, ischemic or mixed ulcer, edema, cyanosis, and gangrene or infection of limbs. Finally, the Rutherford clinical classification was used to stratify patients with severe PAD (Rutherford grades I, II, and III and classes 1 to 6).^25^

Diabetes was defined by the criteria of two fasting glycemia results over 126, glycemia over 200 2 h after a glucose challenge, or casual glycemia over 200 with associated symptoms, whether or not the patient takes insulin, and glycated hemoglobin. Systemic arterial hypertension was defined as systolic arterial blood pressure over 139 mmHg or diastolic arterial blood pressure over 89 mmHg (regardless of treatment), according to the 7th Brazilian Arterial Hypertension Guidelines of 2016,^26^ and renal dysfunction was assessed by creatinine result > normal reference value (1.2 and 1.3 mg/dL for women and men, respectively). Patients’ lipid profiles were defined as total cholesterol (high density lipoprotein [HDL], low density lipoprotein [LDL], very low density lipoprotein, and triglycerides) and vascular physical examination and patient history were used to classify patients’ clinical vascular involvement.^27^

All arteriographies are stored in complete form in DICOM format on the server for the hospital’s digital records system (an MV system). Images were analyzed by an undergraduate scholarship student, always supervised by one of the team’s vascular surgeons. Images were assessed on a workstation, using image optimization tools such as brightness and contrast enhancement, image magnification, digital rulers, and automatic and manual stenosis analyses, among others.

The sample was selected consecutively over the course of the study period, applying the inclusion and exclusion criteria defined. Palpation of pulses, clinical symptoms, and Rutherford classification were the main criteria used when deciding to order angiographs. Although measurements for ABI were taken for these patients, ABI was not included in the study because it is subject to serious limitations in diabetic patients and because we do not routinely calculate the ratio between the pressures at the hallux and arm. Arteriographies confirmed clinical suspicion and were used as the basis for sample selection.

The angiographic images were classified using three different scores: the Inter-Society Consensus for the Management of Peripheral Arterial Disease II (TASC II),^9^ the Bollinger et al. score,28 and a modified Bollinger et al. score.

The TASC II classification is subdivided into the following sectors: aorto-femoral, femoropopliteal, and infrapopliteal. Four types of vascular involvement are scored in each sector, classified as type A - single stenosis; B - mild stenosis or occlusions; C - moderate stenosis or occlusions; and D - extensive stenosis or occlusions.

The aortoiliac and femoropopliteal sectors were classified by the TASC II^29^ score into four classes (TASC II A, B, C, and D).^9^ In the absence of TASC II criteria of arterial injury in a given sector, a value of TASC II = 0 was attributed arbitrarily.

**Figure 1:**
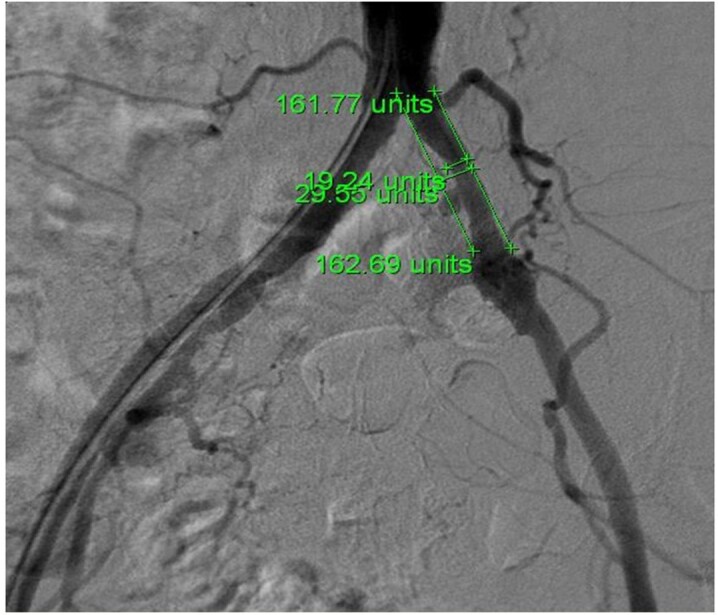
Method used to calculate stenosis for the Bollinger et al. score. Two reference lines were drawn tangential to the lateral arterial walls. Stenosis was measured by comparing the comparative distances between the point of narrowing and the normal reference.

The Bollinger et al. score^28^ was used to conduct angiographic assessments of 10 arteries: 1) abdominal aorta; 2) common iliac artery; 3) external iliac artery (up to the femoral bifurcation); 4) internal iliac artery (up to the first bifurcation); 5) deep femoral artery (the proximal 15 cm of its main branch); 6) superficial femoral artery (up to where it crosses the medial margin of the femur); 7) popliteal artery (up to the bifurcation of the fibular and anterior tibial arteries, excluding the tibial-fibular trunk); 8) anterior tibial artery (up to the proximal 3 cm); 9) fibular artery; and 10) posterior tibial artery (both up to the proximal 5 cm).^29^ The original Bollinger et al. score (Table 1) is a matrix on which occlusions or stenoses (columns) are scored according to the extent of the injury (rows). For example, a > 50% lesion of the adductor canal is scored as 6 points, but if there are also < 25% lesions involving more than half of the artery, a further 3 points are added (total = 9). This score therefore provides a semi-quantitative analysis of the severity of the lesions involving a specific artery in a group of patients and also enables vector analysis (evaluating an arterial segment), which widens the study spectrum (Figure 1).

**Table 1:** Bollinger et al. score table. 1 point is added for occlusions that are longer than 2 cm. Where there is occlusion, other stenoses or plaques are not counted and where > 50% or 25-50% stenosis is present, plaques are not counted.

**Lesion**	**Occlusion**	**Stenosis Plaques**		
**%**	**100%**	**> 50% 25-49% < 25%**		
Single lesion		4	2	1
Multiple lesions affecting less than 50% of the segment	13	5	3	2
Multiple lesions affecting more than 50% of the segment	15	6	4	3

In addition to these scores, an adapted version of the Bollinger et al. score was also created, including scores for the entire distal arterial segment - since the original Bollinger et al. score only covers the proximal 5 cm of the infrapatellar arteries. The authors adopted this modification in order to obtain a score for the entire infrapatellar segment, attributing arbitrary values for the whole of this distal arterial segment, as shown in Table 2. Stenosis classes < 50% were excluded in this segment, because the smaller caliber of the distal arteries prevents assessment of smaller stenoses with the same level of detail as in the proximal arteries. Thus, the arteries of the leg were classified as infrapatellar popliteal artery (segment P3) - up to the emergence of the anterior tibial artery; tibial-fibular trunk - up to the bifurcation of the posterior tibial and fibular arteries; and anterior tibial, fibular, and posterior tibial arteries - all along their entire course, up to the initial formation of the plantar arch (Table 2).

**Table 2:** Modified Bollinger et al. score - proposed scores for the distal sector

**Lesion**	**Score**
Arteries free from plaques	0
Arteries with plaques	1
Arteries with single stenoses	2
Arteries with multiple stenoses	3
Arteries with occlusion or occlusions affecting less than 50% of the segment	5

The arteriographic assessments were conducted using Centricity DICOM Viewer 3.0 software, which is incorporated into the GE Healthcare angiography machine (Figure 1). Scores were allocated by the lead author (GOT), under direct supervision, and confirmed by vascular surgery specialists.

All patients who underwent diagnostic angiography or endovascular intervention with concurrent angiography were analyzed and, as such, the sample was selected by convenience. Since this is a comparative study of patterns of lower limb arterial involvement, a calculation for 95% reliability and 10% margin of error estimated a minimum of 150 patient records, divided between diabetic and non-diabetic groups.

Statistical analysis started with descriptive statistics, calculating frequencies and percentages for qualitative variables and means, medians, standard deviations, and minimum and maximum values for quantitative variables.

To test for associations between the variable DM and explanatory variables of interest, the chi-square test or Fisher’s exact test, when necessary, were used. A generalized linear model with Poisson distribution was used to determine the influence of DM in relation to the sectors and lesions mentioned. For clinical variables, a test of normality was conducted to verify the distribution of data. Student’s *t* test was used to compare means of variables that exhibited symmetrical distribution between groups of diabetic and non-diabetic patients. For variables that exhibited asymmetrical distribution, a generalized linear model with Gamma distribution was estimated. The significance level was set at p < 0.05.

Analyses were performed using SAS version 9.3, by a statistician from the institution’s Research Support Office.

## RESULTS

**Figure 2:**
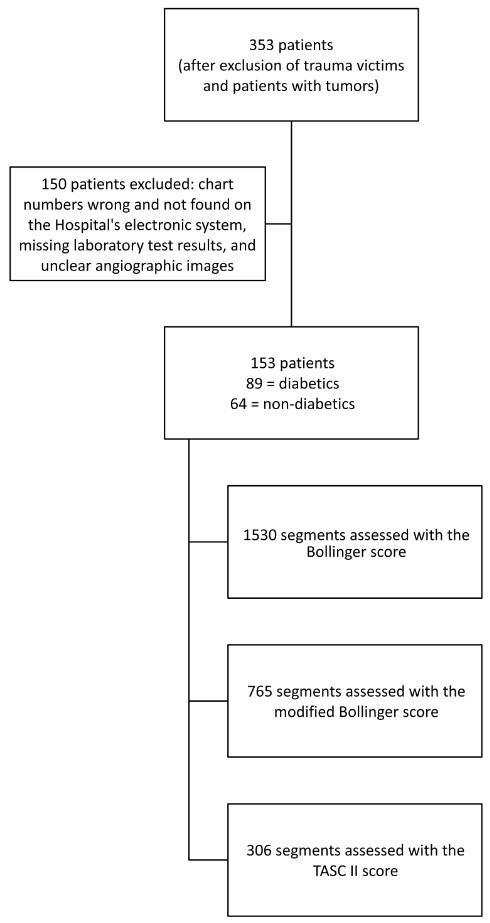
Flow diagram showing recruitment of cases, exclusions, and arterial segments analyzed.

A total of 353 angiographs performed from 2012 to 2016 were initially selected for the study, 200 of which were excluded (n = 153). A total of 1,530 segments were analyzed using the Bollinger et al. score, 306 with the TASC II, and 765 with the modified Bollinger et al. score. Eighty-nine angiographic examinations were of diabetic patients and 64 were of non-diabetic patients (Figure 2).

Demographic results of relevance (Table 3) include a predominance of non-smokers in the group of diabetics (22% vs. 7% p = 0.01). Higher mean HDL laboratory test results were observed in the non-diabetics (40 mg/dL vs. 45 mg/dL; p = 0.001), triglycerides were higher in the diabetics (172 mg/dL vs. 122 mg/dL; p = 0.001), and glycemia was higher in the diabetics (173 mg/dL vs. 93 mg/dL; p = 0.001). Comparison of signs and symptom showed that more diabetics had limb gangrene (10 vs. 1; p = 0.001), limb infection (32 vs. 14; p = 0.02), and personal histories of acute myocardial infarction (18 vs. 3; p = 0.03) and stroke (12 vs. 2; p = 0.02). The comparisons of age, sex, SAH and, smoking load were not statistically significant between the two groups. Rutherford classes 5 and 6 were more frequent among the diabetics (p = 0.018), but the frequency of classes 3 and 4 was similar in both groups (p = 0.244).

**Table 3:** Demographic data and risk factors, comparison between groups.

**Variables**		Diabetics*	**Non diabetics**	**Total**	***P***
Sex	Male	40 (26%)	35 (22%)	75	0.23
	Female	49 (32%)	29 (18%)	78	
Ethnicity	Mixed race	4 (2%)	10 (6%)	14	0.04
	Black	6 (3%)	2 (1%)	8	
	White	79 (51%)	52 (33%)	131	
Smoking	Non-smoker	34 (22%)	11 (7%)	45	0.01
	Smoker	29 (18%)	25 (16%)	54	
	Ex-smoker	26 (16%)	28 (17%)	54	
Tobacco load (pack-years)		40	38		0.70
SAH	Not hypertensive	13 (8%)	15 (9%)	28	0.2
	Hypertensive	69 (45%)	42 (27%)	111	
	Untreated hypertensive	7 (4%)	7 (4%)	14	
Creatinine (mg/dL)		1.07	1		0.39
Urea (mg/dL)		39	40		0.73
HDL (mg/dL)		40	45		0.001
Total cholesterol (mg/dL)		166	157		0.22
Triglycerides (mg/dL)		172	122		0.001
Glycemia (mg/dL)		173	93		0.001
Age (years)		66	68		0.5
Cyanosis of limbs		25 (16%)	22 (14%)	47	0.09
Edema of limbs		17 (11%)	7 (4%)	24	0.07
Rutherford	3	9 (5%)	15 (9%)	24 (15%)	0.244
	4	20 (13%)	18 (11%)	38 (26%)	
	5	41 (26%)	25 (16%)	66 (43%)	0.018
	6	19 (12%)	6 (3%)	25 (16%)	
	Total	89	64	153	0.038
Gangrene of limbs		10 (6%)	1 (0.6%)	11	0.01
Infection of limbs		32 (20%)	14 (9%)	46	0.02
Ischemic ulcer		48 (31%)	28 (18%)	76	0.06
Mixed ulcer		14 (9%)	5 (3%)	19	0.07
Pain at rest		37 (24%)	34(22%)	71	0.40
Stable angina		2 (1%)	0	2	0.22
Prior AMI		18 (11%)	6 (3%)	24	0.03
Prior stroke		12 (7%)	2 (1%)	14	0.02
Total		89 (58%)	64 (41%)	153	

AMI: acute myocardial infarction. * = p < 0.05.

**Figure 3:**
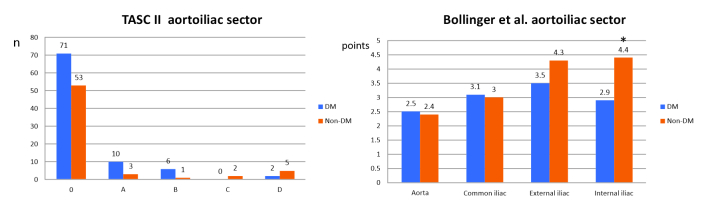
Comparison of results for aortoiliac sector between diabetics and non-diabetics using the TASCII score and the Bollinger et al. score, by arteries analyzed. *= p < 0.05

In the aortoiliac sector (Figure 3), mean total Bollinger et al. scores (stenosis + occlusions) were significantly higher in non-diabetics for the internal iliac artery only (mean scores of 4.4 vs. 2.9 points; p = 0.001). In turn, the TASC II assessment of the aortoiliac sector revealed no significant differences between the two groups (p = 0.051), although the difference was close to attaining significance.

**Figure 4:**
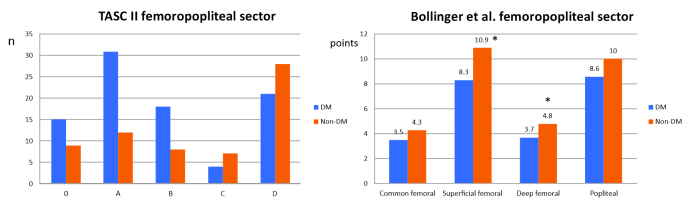
Comparison of results for the sector between diabetics and non-diabetics using the TASCII score and the Bollinger et al. score, by arteries analyzed. *= p < 0.05.

In the femoropopliteal sector (Figure 4), non-diabetics had higher mean total Bollinger et al. scores for the deep femoral artery (mean score of 3.7 vs. 4.8; p = 0.04) and the superficial femoral artery (mean score of 8.3 vs. 10.9; p = 0.008). In turn, the TASC II assessment revealed a higher frequency of severe infrapatellar classifications (TASC II C and D) among non-diabetics than diabetics (p = 0.019).

**Figure 5:**
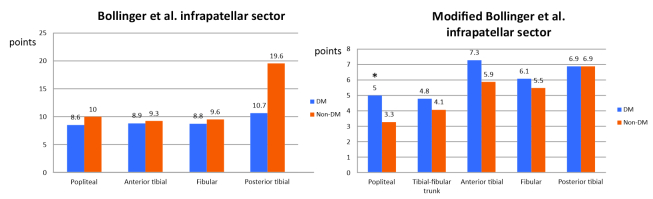
Results for the Bollinger et al. and modified Bollinger et al. scores for the infrapatellar sector between diabetics and non-diabetics, by arteries analyzed. *= p < 0.05.

In the infrapatellar sector (Figure 5), the Bollinger et al. score showed means for > 50% stenosis in the anterior tibial artery (mean scores of 0.7 vs. 0.4; p = 0.005) and 25-50% in the fibular arteries (mean score of 0.8 vs. 0.5; p = 0.04) and posterior tibial arteries (mean score of 0.5 vs. 0.3; p = 0.04) in the diabetic patients, but with no significant differences in the total scores for these arteries. Figure 5 shows the total scores for each artery, summing stenoses and occlusions.

The only statistically significant difference in the modified Bollinger et al. scores (Figure 5) was for popliteal involvement, which was more accentuated among diabetic patients (mean score of 5.0 vs. 3.3; p = 0.02). The anterior tibial artery scored higher among diabetics, but without statistical significance (p = 0.06).

## DISCUSSION

With regard to the objective of this study, in general, distal arterial involvement was more frequent among the diabetics and proximal lesions were more frequent among non-diabetics. Among the principal risk factors, smoking was less frequent among diabetics, dyslipidemia was more frequent among diabetics, and SAH was similar in both groups.

Although analysis of ethnicity was not one of the study objectives, it was observed that there was a significant predominance of white patients in both groups.^30^^-^ 32 The proportions of certain types and ethnicities and their relationship with cardiovascular diseases vary greatly by continent and region.^32^^,^ 33

Smoking was less frequent among diabetic patients, but the tobacco load of smokers and ex-smokers was similar. These data show that smoking was not of itself an important risk factor for development of PAD in the patient sample as a whole. The association between smoking and DM, SAH, and dyslipidemia is cumulative in development of vascular disease.^9^ This study is in agreement with data in the literature in terms of demographic data. Nonetheless, Santos et al.^18^ found a larger proportion of smokers in a non-diabetic group, which is a subset that needs greater attention to lifestyle changes and because smoking is an independent risk factor for development of PAD.

Dyslipidemia was more frequent among diabetics (HDL cholesterol and triglycerides). However, all patients with PAD treated at this center are given prescriptions for statins, irrespective of whether they have some type of dyslipidemia, which could interfere with this type of analysis. The present study was also unable to determine the patients’ degree of compliance with their medication. In general, dyslipidemia in diabetics tends to be characterized by increased triglycerides and reduced HDL cholesterol, while LDL cholesterol concentrations generally do not differ between diabetics and non-diabetics.^30^ When they studied the morphology of atherosclerotic plaques in diabetics and non-diabetics, He et al.^31^ also found that the diabetics had significantly higher levels of triglycerides and, obviously, of glycemia. In contrast to the present study, however, total cholesterol was also elevated in the diabetics, although HDL levels were similar. All of these data are in line with the systemic nature of involvement in DM, causing imbalances in the lipid profile that are most obvious in regard to triglycerides.^32^

Infections and wet gangrene tend to be more common in diabetic patients,^16^^,^ 33 especially in our setting, because of access problems, low socioeconomic status, and lack of information,^34^ making these patients more susceptible to developing these injuries.^16^ In this study, data on these conditions are merely for reference, because the study is focused on arterial damage.

The aortoiliac TASC II scores revealed no significant differences between the two groups. This sector encompasses larger caliber arteries and it is possible that the calcium deposits that are more common among diabetics could have provoked greater local patency. This analysis may not have been significant because there really was no difference between the groups or possibly because the sample was not large enough to detect differences. In general, the Bollinger et al. score for this sector is similar to the TASC II, but it is a little more sensitive for the internal iliac artery, with higher scores (significant) among the non-diabetics. We do not see any explanation for this peculiarity. Few previous studies have conducted comparative assessments of this sector in diabetics and non-diabetics, since the incidence of lesions in these sites is low, which makes it difficult to conduct precise statistical assessments.

Previous studies have shown more severe arterial involvement in the femoropopliteal than the aortoiliac sector in both groups,^18^^,^ 19 ^,^ 23 ^,^ 24 ^,^ 35 with a higher frequency of occlusions than of stenoses. This characteristic was observed in studies by Graziani et al.^23^ (who only investigated diabetics) and Bradbury et al.^35^ (who only investigated non-diabetics), while both studies revealed higher frequencies of severe stenoses and occlusions in this sector, although with an even higher frequency in the infrapatellar sector. In turn, a comparative angiographic study of the two groups^18^ observed that a sample of 117 patients (87 diabetics and 74 non-diabetics) with femoropopliteal occlusion exhibited no difference between diabetics and non-diabetics in terms of popliteal artery refilling. In the present study, the TASC II score for the femoropopliteal sector showed a higher frequency of severe classifications (TASC II C and D) among non-diabetics than among diabetics, which further confirms the statement that non-diabetics exhibit more accentuated proximal involvement than distal, in contrast to what is seen in diabetics. The Bollinger et al. score also showed a predominance of lesions in deep femoral, superficial femoral, and popliteal arteries among non-diabetics. Arterial narrowing in the adductor canal and arterial encapsulation inside this muscular aponeurotic canal have been identified as factors predisposing to plaques in this area.^36^ However, there is no explanation for the fact that diabetics exhibit different behavior in this sector.

Previous studies^18^^,^ 19 ^,^ 23 ^,^ 24 ^,^ 35 have also reported a greater frequency of lesions in the infrapatellar sector in diabetic patients. The present study corroborates these findings with a significantly higher frequency of > 50% stenosis in the anterior tibial artery and 25-50% stenosis in the fibular and posterior tibial arteries. However, total scores (stenosis + occlusions) were similar in both groups (Figure 4). With relation to distal involvement, Santos et al.^18^ found a similar frequency of opacification of popliteal, fibular, and anterior tibial arteries in diabetics and non-diabetics, although there was a higher frequency of posterior tibial artery occlusion among diabetics (p = 0.008). In contrast, the modified Bollinger et al. score revealed greater popliteal involvement among the diabetics. Additionally, in a study by Santos et al.,^18^ the diabetics were predominantly female, hypertensive, and non-smokers, and logistic regression showed that only female sex was a risk factor for non-opacification of the posterior tibial artery. In an analogous study, Jude et al.^24^ showed that arterial injuries in diabetics were predominantly in the deep femoral artery and all infrapatellar arteries, but these authors did not conduct qualitative stratification of the injuries found. Thus, in the present study, presence of stenosis in the three major arteries of the leg was significant, but there was only a significant association with > 50% stenosis for the anterior tibial artery. Multiple lesions in distal arteries can limit the success of vascular reconstruction because of deficient runoff.^37^

Certain limitations of the present study should be acknowledged. It is a retrospective study and, as such, patient records tend to have a considerable proportion of missing data, which leads to a large number of exclusions and a high probability of biases, thereby weakening the quality of the sample and the study conclusions. Another limitation was the inability to analyze damage to the extremities using the Wagner, WIFi, or Glass classifications,^38^ also because of the study’s retrospective design. Although scores were used, and checked by specialists, a certain degree of subjectivity may have affected assessment of the arteriographies. The dyslipidemia data may be subject to bias because the patients were taking statins. Non-significant results may have been because of true absence of significance or because the sample was too small after the excessive losses. A large-scale multicenter sample could clear up this doubt.

Randomized prospective studies, and particularly multicenter designs, are needed to improve the quality of evidence on the differential profile of peripheral artery involvement in diabetics.

## CONCLUSIONS

Among the patients in the present study, the Bollinger et al. score revealed higher scores for arterial lesions to the internal iliac, deep femoral, and superficial femoral arteries among the non-diabetics. However, popliteal artery scores were higher among diabetics. The TASC II classification revealed similar frequencies of aortoiliac classifications in diabetics and non-diabetics and higher frequencies of C and D classifications for the femoropopliteal sector among non-diabetics.

In the infrapatellar segment, the Bollinger et al. stenosis scores were significantly higher among the diabetics for all three distal arteries (anterior tibial, posterior tibial, and fibular), but there was no difference in total score (occlusions + stenosis). The modified Bollinger et al. scores were only different for the popliteal artery, with greater involvement among the diabetics.

## References

[1] Chacra APM, Santos RD, Maffei F (2015). Doenças vasculares periféricas.

[2] Abbas AK, Fausto N, Kumar V (2018). Robbins Patologia Básica.

[3] Durazzo AES, Sitrângulo CJ, Presti C, Silva ES, De Luccia N (2005). Doença arterial obstrutiva periférica: que atenção temos dispensado à abordagem clínica dos pacientes?. J Vasc Bras.

[4] Roth GA, Forouzanfar MH, Moran AE (2015). Demographic and epidemiologic drivers of global cardiovascular mortality. N Engl J Med.

[5] Mansur AP, Favarato D (2012). Mortalidade por doenças cardiovasculares no Brasil e na região metropolitana de São Paulo: atualização 2011. Arq Bras Cardiol.

[6] Jesus-Silva SG, Oliveira JP, Brianezi MHC, Silva MAM, Krupa AE, Cardoso RS (2017). Análise dos fatores de risco relacionados às amputações maiores e menores de membros inferiores em hospital terciário. J Vasc Bras.

[7] Yoshida RA, Matida CK, Sobreira ML (2008). Estudo comparativo da evolução e sobrevida de pacientes com claudicação intermitente, com ou sem limitação para exercícios, acompanhados em ambulatório específico. J Vasc Bras.

[8] Welter HF, Kettmann R, Grothe A (2002). Peripheral arterial occlusive disease. Symptoms, basic diagnosis and staged therapy. MMW Fortschr Med.

[9] Norgren L, Hiatt WR, Dormandy JA (2007). Inter-Society Consensus for the Management of Peripheral Arterial Disease (TASC II). J Vasc Surg.

[10] Leite CF, Frankini AD, DeDavid EB (2004). Análise retrospectiva sobre a prevalência de amputações bilaterais de membros inferiores. J Vasc Bras.

[11] Silva LR, Fernandes GM, Morales NU (2018). Results of One-Stage or Staged Amputations of Lower Limbs Consequent to Critical Limb Ischemia and Infection. Ann Vasc Surg.

[12] Nunes MAP, Resende KF, Castro AA, Pitta GBB, Figueiredo LFP, Miranda F (2006). Fatores predisponentes para amputação de membro inferior em pacientes diabéticos internados com pés ulcerados no estado de Sergipe. J Vasc Bras.

[13] Santos ICRV, Carvalho EF, Souza WV, Albuquerque EC (2015). Factors associated with diabetic foot amputations. J Vasc Bras.

[14] Dos Santos VP, da Silveira DR, Caffaro RA (2006). Risk factors for primary major amputation in diabetic patients. Sao Paulo Med J.

[15] Senefonte FRA, Rosa GRPS, Comparin ML (2012). Amputação primária no trauma: perfil de um hospital da região centro-oeste do Brasil. J Vasc Bras.

[16] Cardoso NA, Cisneros LL, Machado CJ, Procópio RJ, Navarro TP (2018). Risk factors for mortality among patients undergoing major amputations due to infected diabetic feet. J Vasc Bras.

[17] De Luccia N (2003). Doença vascular e diabetes. J Vasc Bras.

[18] Santos VP, Alves CA, Fidelis C, Araújo JS (2013). Arteriographic findings in diabetic and non-diabetic with critical limb ischemia. Rev Assoc Med Bras (1992).

[19] Santos VP, Caffaro RA, Pozzan G, Saieg MA, Castelli V (2008). Comparative histological study of atherosclerotic lesions and microvascular changes in amputated lower limbs of diabetic and non-diabetic patients. Arq Bras Endocrinol Metabol.

[20] Brazeau NF, Pinto EG, Harvey HB (2013). Critical limb ischemia: an update for interventional radiologists. Diagn Interv Radiol.

[21] Pomposelli F (2010). Arterial imaging in patients with lower extremity ischemia and diabetes mellitus. J Vasc Surg.

[22] Rueda CA, Nehler MR, Perry DJ (2008). Patterns of artery disease in 450 patients undergoing revascularization for critical limb ischemia: implications for clinical trial design. J Vasc Surg.

[23] Graziani L, Silvestro A, Bertone V (2007). Vascular involvement in diabetic subjects with ischemic foot ulcer: a new morphologic categorization of disease severity. Eur J Vasc Endovasc Surg.

[24] Jude EB, Oyibo SO, Chalmers N, Boulton AJ (2001). Peripheral arterial disease in diabetic and nondiabetic patients: a comparison of severity and outcome. Diabetes Care.

[25] Rutherford RB, Baker JD, Ernst C (1997). Recommended standards for reports dealing with lower extremity ischemia: revised version. J Vasc Surg.

[26] Malachias M, Plavnik FL, Machado CA, Malta D, Scala LCN, Fuchs S (2016). 7ª Diretriz Brasileira de Hipertensão Arterial: Capítulo 1-Conceituação, Epidemiologia e Prevenção Primária. Arq Bras Cardiol.

[27] Zhu C, Zhou B, Lu J (2019). Principles of STAGE Management for Diabetic Foot Ulcers Based on the Wagner and Texas Classification Systems. Int J Low Extrem Wounds.

[28] Bollinger A, Breddin K, Hess H (1981). Semiquantitative assessment of lower limb atherosclerosis from routine angiographic images. Atherosclerosis.

[29] Stoner MC, Calligaro KD, Chaer RA (2016). Reporting standards of the Society for Vascular Surgery for endovascular treatment of chronic lower extremity peripheral artery disease. J Vasc Surg.

[30] Rodgers A, Ezzati M, Vander Hoorn S, Lopez AD, Lin RB, Murray CJ (2004). Distribution of major health risks: findings from the Global Burden of Disease study. PLoS Med.

[31] He C, Yang ZG, Chu ZG (2010). Carotid and cerebrovascular disease in symptomatic patients with type 2 diabetes: assessment of prevalence and plaque morphology by dual-source computed tomography angiography. Cardiovasc Diabetol.

[32] Zhou M, Zhu L, Cui X (2016). The triglyceride to high-density lipoprotein cholesterol (TG/HDL-C) ratio as a predictor of insulin resistance but not of beta cell function in a Chinese population with different glucose tolerance status. Lipids Health Dis.

[33] Mills JL, Conte MS, Armstrong DG (2014). he Society for Vascular Surgery Lower Extremity Threatened Limb Classification System: risk stratification based on wound, ischemia, and foot infection (WIfI). J Vasc Surg.

[34] Berlanga-Acosta J, Schultz GS, Lopez-Mola E, Guillen-Nieto G, García-Siverio M, Herrera-Martínez L (2013). Glucose toxic effects on granulation tissue productive cells: the diabetics’ impaired healing. BioMed Res Int.

[35] Bradbury AW, Adam DJ, Bell J (2010). Bypass versus Angioplasty in Severe Ischaemia of the Leg (BASIL) trial: a description of the severity and extent of disease using the Bollinger angiogram scoring method and the TransAtlantic Inter-Society Consensus II classification. J Vasc Surg.

[36] Han Y, Zhu Z, Guan M (2020). Diabetes-specific characteristics of atherosclerotic plaques in femoral arteries determined by three-dimensional magnetic resonance vessel wall imaging. Diabetes Metab Res Rev.

[37] Yoshida RA, Silva CEC, Sobreira ML, Yoshida WB (2008). Angioplastia infrapoplítea: quanto mais artérias tratar, melhor?. J Vasc Bras.

[38] Conte MS, Bradbury AW, Kolh P (2019). Global vascular guidelines on the management of chronic limb-threatening ischemia. J Vasc Surg.

