# Supplementary material for: Comparative study of angiographic changes in diabetic and non-diabetic patients with peripheral arterial disease
Source: J Vasc Bras. 2023 Feb 10;22:e20200053. doi: 10.1590/1677-5449.202000532 (PMC9925060; doi:10.1590/1677-5449.202000532)
Supplement: Translation

# Translation: Comparação das alterações angiográficas entre pacientes diabéticos e não diabéticos com doença arterial periférica

**DOI:** 10.1590/1677-5449.202000531

**Authors:** Giovanni Ortale Trainotti, Jamil Victor Mariúba, Matheus Bertanha, Marcone Lima Sobreira, Ricardo de Alvarenga Yoshida, Rodrigo Gibin Jaldin, Paula Angeleli Bueno de Camargo, Winston Bonetti Yoshida

**Affiliations:** 1 Universidade Estadual Paulista - UNESP, Faculdade de Medicina de Botucatu, Hospital das Clínicas, Botucatu, SP, Brasil.

**Keywords:** angiografia digital, doenças vasculares periféricas, diabetes

## Abstract

**Contexto:**

Os diabéticos possuem risco de 5 a 15 vezes maior para o desenvolvimento de doença arterial periférica (DAP), e poucos estudos compararam fatores de risco e a distribuição e gravidade de alterações arteriais angiográficas entre diabéticos e não diabéticos.

**Objetivos:**

Comparar alterações angiográficas entre pacientes diabéticos e não diabéticos com DAP avançada, correlacionando-as com demais fatores de risco.

**Métodos:**

Trata-se de um estudo transversal retrospectivo de pacientes consecutivos submetidos a arteriografia de membros inferiores por DAP (Rutherford de 3 a 6), usando os escores angiográficos TASC II e de Bollinger et al. Os critérios de exclusão incluíram arteriografias de membros superiores, exames incompletos ou sem nitidez e cirurgias prévias. A análise estatística incluiu o teste do qui-quadrado ou exato de Fisher para variáveis discretas e o teste *t* para variáveis contínuas (significância: p < 0,05).

**Resultados:**

Foram estudados 153 pacientes com idade média de 67 anos, sendo 50,9% do sexo feminino e 58,2% diabéticos. Um total de 91 pacientes (59%) tinha lesão trófica (Rutherford 5 ou 6), enquanto 62 (41%) tinham dor em repouso ou claudicação limitante (Rutherford 3 e 4). Entre os diabéticos, 81,7% eram hipertensos, 29,4% nunca fumaram e 14% tinham antecedente de infarto do miocárdio. Pelo escore de Bollinger et al., as artérias infrapoplíteas foram as mais comprometidas, em especial a tibial anterior (p = 0,005) nos diabéticos, enquanto a femoral superficial foi mais acometida nos não diabéticos (p = 0,008). Pelo TASC II, as alterações arteriográficas mais graves ocorreram no segmento fêmoro-poplíteo nos pacientes não diabéticos (p = 0,019).

**Conclusões:**

Os setores infrapoplíteos foram os mais comprometidos nos diabéticos, enquanto o setor femoral foi o mais acometido nos não diabéticos.

## INTRODUÇÃO

A aterosclerose é a principal causa de doenças cardiovasculares^1^^,^ 2, grupo que engloba doença arterial coronariana, doença arterial periférica (DAP) e doença cerebrovascular^3^. Se todas as manifestações fossem consideradas entidade patológica única, a aterosclerose seria considerada a principal causa de morte no mundo, estimada em 1/3 de todas as mortes mundiais4, e no Brasil^5^.

Hipertensão arterial sistêmica (HAS), diabetes melito (DM) e tabagismo são grandes agravantes da degeneração da parede arterial^1^^-^ 3 ^,^ 6, e o controle dessas condições tem forte influência sobre o prognóstico do paciente^7^^-^ 9.

A DAP acomete principalmente pacientes idosos com fatores de risco para aterosclerose^6^^,^ 9 e se manifesta insidiosamente por claudicação intermitente progressiva^7^. O desenvolvimento de dor ao repouso, úlceras isquêmicas e/ou gangrena caracteriza a isquemia crítica do membro (ICM), que ocorre em cerca de 5-10% dos casos^9^. A melhor opção terapêutica para esses casos costuma ser a revascularização do membro, exceto em pacientes com graves comorbidades ou prognóstico muito limitado de sucesso de revascularização, para os quais a amputação passa a ser o tratamento mais apropriado^10^^-^ 15.

Mais da metade dos pacientes com DAP apresenta índice tornozelo-braço (ITB) < 0,9 e é assintomática. A cada 100 pacientes com claudicação intermitente, 25% evoluem para piora da claudicação, 5-10% são submetidos a cirurgia de revascularização, 2-5% são submetidos a amputação e cerca de 30% evoluem para o óbito^9^^,^ 13 ^,^ 16. O risco de os diabéticos desenvolverem DAP é de 5 a 15 vezes maior em relação aos não diabéticos^12^^,^ 14 ^,^ 17. Nos casos com ICM, é recomendável o estudo arterial de imagem para melhor caracterização da lesão e programação cirúrgica. A arteriografia é o exame complementar diagnóstico de escolha para avaliação da circulação arterial periférica^18^^-^ 21.

Os poucos estudos que compararam as lesões arteriais de pacientes portadores de DAP e DM mostraram que, nos diabéticos, as lesões arteriais costumam se concentrar nas artérias de menor calibre (infrapoplíteas)^14^^,^ 18 ^,^ 19 ^,^ 22 ^-^ 24. Porém, nenhum dos estudos utilizou escores angiográficos para classificar as lesões e as correlacionar com fatores de risco e dados demográficos.

Nosso objetivo, portanto, foi quantificar e comparar por meio de escores as alterações angiográficas de pacientes diabéticos e não diabéticos com DAP avançada, correlacionando-as com demais fatores de risco.

## MATERIAIS E MÉTODOS

Realizou-se um estudo observacional, retrospectivo e transversal de série consecutiva de casos, com análise de arteriografias realizadas de 2012 até dezembro de 2016 em uma única instituição, comparando pacientes diabéticos e não diabéticos. O estudo foi aprovado pelo Comitê de Ética local (parecer 1.578.037).

Foram incluídos pacientes consecutivos com DAP avançada (Rutherford 3 a 6), diabéticos e não diabéticos. Resumidamente, a classificação de Rutherford compreende as seguintes categorias^25^: 0 = assintomático; 1 = claudicação leve; 2 = claudicação moderada; 3 = claudicação grave; 4 = dor em repouso; 5 = lesão trófica pequena; 6 = necrose extensa. As categorias de 3 a 6 foram arbitrariamente consideradas como avançadas no presente estudo. Os critérios de exclusão incluíram angiografias de membros superiores, pacientes que já haviam sido submetidos a intervenções cirúrgicas de revascularização, rol de exames laboratoriais incompleto e angiografias sem nitidez.

A coleta de dados demográficos foi feita no sistema de prontuário eletrônico MV do hospital e incluiu idade, etnia autodeclarada no registro do prontuário, comorbidades e exames laboratoriais (ureia, creatinina, glicemia e perfil lipídico). As queixas clínicas referidas pelos pacientes e conferidas pelo médico foram as seguintes: dor ao repouso, úlcera isquêmica ou mista, edema, cianose, gangrena ou infecção de membros. Por fim, utilizou-se a classificação clínica de Rutherford para estratificar os pacientes com DAP grave (classificação de Rutherford em graus I, II e III e classes de 1 a 6)^25^.

O DM foi definido pelo critério de duas glicemias em jejum acima de 126, glicemia 2 h após sobrecarga de glicose acima de 200 ou glicemia casual acima de 200 com sintomas associados, usuário de insulina ou não, e hemoglobina glicada. A HAS foi definida como pressão arterial sistólica acima de 139 mmHg ou pressão arterial diastólica acima de 89 mmHg (tratada ou não), de acordo com a 7ª Diretriz Brasileira de Hipertensão Arterial de 201626, e alteração da função renal pelo valor da creatinina > valor de referência (1,2 e 1,3 mg/dL para mulheres e homens, respectivamente). O colesterol total (lipoproteína de alta densidade [HDL], lipoproteína de baixa densidade [LDL], lipoproteína de muito baixa densidade e triglicérides) definiu o perfil lipídico do paciente, e anamnese e exame físico vascular foram usados para classificar o acometimento clínico vascular dos pacientes^27^.

Todas as arteriografias ficam arquivadas de forma completa e integral em formato DICOM no servidor do sistema digital do hospital (sistema MV). As imagens foram analisadas por um aluno de iniciação científica, sempre sob a supervisão de um dos cirurgiões vasculares da equipe. As imagens foram avaliadas em estação de trabalho, usando ferramentas de otimização de imagens, como realce de nitidez e contraste, magnificação das imagens, réguas digitais, análises automáticas e manuais de estenoses, entre outras.

A amostra foi selecionada de forma consecutiva no período do estudo, conforme os critérios de inclusão e exclusão discriminados. A palpação de pulsos, os sintomas clínicos e a categorização de Rutherford foram os principais critérios para indicação de angiografias. Embora sejam realizadas medidas de ITB nesses pacientes, o ITB não foi incluído por apresentar grandes limitações em pacientes diabéticos e por não realizarmos como rotina medidas do índice de pressão entre as pressões do hálux e do braço. As arteriografias confirmaram a suspeita clínica e serviram de base para a constituição da amostra.

As imagens angiográficas foram classificadas por meio de três escores: *Inter-Society Consensus for the Management of Peripheral Arterial Disease II* (TASC II)^9^, Bollinger et al.^28^ e Bollinger et al. modificado.

A classificação TASC II é dividida nos seguintes setores: aorto-femoral, fêmoro-poplítea e infrapoplítea. Em cada setor, são discriminados quatro tipos de acometimento vascular, classificados em padrão A - estenoses pontuais; B - estenoses ou oclusões leves; C - estenoses ou oclusões moderadas; e D - estenoses ou oclusões extensas.

Os setores aorto-ilíaco e fêmoro-poplíteo foram classificados pelo score TASC II^29^ em quatro classes (TASC II A, B, C e D)^9^. Na ausência de critério TASC II de lesão arterial em determinado setor, foi atribuído arbitrariamente o valor TASC II = 0.

O escore de Bollinger et al.^28^ foi utilizado para realizar a avaliação angiográfica de 10 artérias: 1) aorta abdominal; 2) artéria ilíaca comum; 3) artéria ilíaca externa (até a bifurcação femoral); 4) artéria ilíaca interna (até a primeira bifurcação); 5) artéria femoral profunda (15 cm proximais de seu principal ramo); 6) artéria femoral superficial (até o seu cruzamento pela borda medial do fêmur); 7) artéria poplítea (até a bifurcação fibular e tibial anterior, excluindo o tronco tíbio-fibular); 8) artéria tibial anterior (até 3 cm proximais); 9) artéria fibular; e 10) artéria tibial posterior (ambas até os 5 cm proximais)^29^. O escore de Bollinger et al. original (Tabela 1) é uma matriz em que oclusão ou estenoses são pontuadas (colunas) conforme a extensão da lesão (linhas). Por exemplo, uma lesão > 50% no canal dos adutores recebe 6 pontos, mas, se também apresentar lesões < 25% em mais de metade da artéria, são adicionados mais 3 pontos (total = 9). Assim, esse escore permite uma avaliação semiquantitativa da gravidade das lesões de uma artéria específica em um grupo de pacientes, bem como uma análise vetorial (permitindo a avaliação de um segmento arterial), o que amplia o espectro do estudo (Figura 1).

**Tabela 1:** Pontuação de acordo com o escore de Bollinger et al. Soma-se 1 ponto às oclusões caso elas ultrapassem 2 cm de extensão. Nas oclusões, outras estenoses ou placas presentes são desconsideradas e, na presença de estenoses > 50% e de 25-50%, as placas são desconsideradas.

**Lesão**	**Oclusão**	**Estenoses Placas**		
**%**	**100%**	**> 50% 25-49% < 25%**		
Lesão simples		4	2	1
Lesões múltiplas afetando menos de 50% do segmento	13	5	3	2
Lesões múltiplas afetando mais de 50% do segmento	15	6	4	3

**Figura 1:** Método usado para calcular a estenose pelo escore de Bollinger et al. Foram traçadas duas linhas de referência tangenciando as paredes laterais arteriais. As medidas das estenoses foram feitas pelas distâncias comparativas entre o estreitamento e a referência normal.

Além desses escores, foi feita uma adaptação do escore de Bollinger et al., incluindo a pontuação de todo o segmento arterial distal - visto que o escore de Bollinger et al. abrange somente os 5 cm proximais das artérias infrapatelares. A modificação foi introduzida pelos autores para pontuar todo o segmento infrapatelar, atribuindo valores arbitrários em todo esse segmento arterial distal conforme a Tabela 2. As classes de estenoses < 50% foram excluídas nesse setor, pois as artérias distais, por serem de pequeno calibre, impossibilitam avaliações de estenoses menores com o mesmo grau de detalhamento que as artérias proximais. Dessa forma, as artérias da perna foram classificadas em artéria poplítea infrapatelar (segmento P3) - até a emergência da artéria tibial anterior -, tronco tíbio-fibular - até a bifurcação em artérias tibial posterior e fibular -, seguido das artérias tibial anterior, fibular e tibial posterior - todas em todo o seu segmento, até o início da formação do arco plantar (Tabela 2).

**Tabela 2:** Escore de Bollinger et al. modificado - pontuação proposta para o setor distal

**Lesão**	**Pontuação**
Aa. sem placas	0
Aa. com placas	1
Aa. com estenose simples	2
Aa. com estenose múltipla	3
Aa. com oclusão ou oclusões afetando até 50% do segmento	5

A avaliação arteriográfica foi feita por meio do programa Centricity DICOM Viewer 3.0, incorporado ao aparelho de angiografia GE Healthcare (Figura 1). Os escores foram obtidos pelo autor (GOT), sob supervisão direta, e conferidos por especialistas em cirurgia vascular.

Foram analisados todos os pacientes submetidos a angiografia diagnóstica ou intervenção endovascular com angiografia concomitante e, portanto, a amostra foi de conveniência. Tratando-se de um estudo comparativo dos padrões de acometimento arterial de extremidades inferiores, calculou-se com confiabilidade de 95% e margem de erro de 10% a consulta de no mínimo 150 prontuários, divididos em grupos de diabéticos e não diabéticos.

Para a análise estatística, foi realizada estatística descritiva com frequências e porcentagens para as variáveis qualitativas e com média, mediana, desvio padrão e valores mínimo e máximo para as variáveis quantitativas.

Para verificar a associação entre a variável DM e as variáveis explanatórias de interesse, foi utilizado o teste do qui-quadrado ou exato de Fisher, quando necessário. Para verificar a influência do DM em relação aos setores e lesões mencionados, foi ajustado um modelo linear generalizado com distribuição de Poisson. Para as variáveis clínicas, foi realizado um teste de normalidade para verificar a distribuição dos dados; para as variáveis que apresentaram uma distribuição simétrica, realizou-se o teste *t* de Student para comparar as médias entre os grupos de pacientes diabéticos e não diabéticos. Para os que apresentaram uma distribuição assimétrica, ajustou-se um modelo linear generalizado com distribuição Gama. Considerou-se p < 0,05 como nível de significância.

As análises foram feitas por meio do programa SAS, versão 9.3, por estaticista do Escritório de Apoio à Pesquisa da instituição.

## RESULTADOS

No período de 2012 a 2016, foram selecionadas 353 angiografias para o estudo, das quais foram excluídas 200 (n = 153). No total, foram analisados 1.530 segmentos pelo escore de Bollinger et al., 306 pelo TASC II e 765 pelo escore de Bollinger et al. modificado. Dos exames angiográficos, 89 eram de pacientes diabéticos e 64, de não diabéticos (Figura 2).

**Figura 2:** Fluxograma mostrando o recrutamento de casos, as exclusões e os segmentos arteriais analisados.

Entre os resultados demográficos relevantes (Tabela 3), observou-se predomínio de não tabagistas no grupo dos diabéticos (22% vs. 7% p = 0,01). Foram observados maiores valores laboratoriais médios de HDL em não diabéticos (40 mg/dL vs. 45 mg/dL; p = 0,001), de triglicérides em diabéticos (172 mg/dL vs. 122 mg/dL; p = 0,001) e de glicemia em diabéticos (173 mg/dL vs. 93 mg/dL; p = 0,001). Já entre as variáveis semiológicas, houve mais diabéticos com gangrena de membros (10 vs. 1; p = 0,001), infecção de membros (32 vs. 14; p = 0,02) e antecedentes pessoais de infarto agudo do miocárdio (18 vs. 3; p = 0,03) e acidente vascular cerebral (12 vs. 2; p = 0,02). A comparação entre as variáveis idade, sexo, HAS e carga tabágica não foi estatisticamente significativa entre os dois grupos. As classes de Rutherford 5 e 6 foram mais frequentes nos diabéticos (p = 0,018), mas as frequências das classes 3 e 4 foram similares entre os dois grupos (p = 0,244).

**Tabela 3:** Dados demográficos e fatores de risco comparativos entre os dois grupos.

**Variáveis**		Diabéticos*	**Não diabéticos**	**Total**	***P***
Sexo	Masculino	40 (26%)	35 (22%)	75	0,23
	Feminino	49 (32%)	29 (18%)	78	
Etnia	Mulato	4 (2%)	10 (6%)	14	0,04
	Negro	6 (3%)	2 (1%)	8	
	Branco	79 (51%)	52 (33%)	131	
Tabagismo	Não tabagista	34 (22%)	11 (7%)	45	0,01
	Tabagista	29 (18%)	25 (16%)	54	
	Ex-tabagista	26 (16%)	28 (17%)	54	
Carga tabágica (anos-maço)		40	38		0,70
HAS	Não hipertenso	13 (8%)	15 (9%)	28	0,2
	Hipertenso	69 (45%)	42 (27%)	111	
	Hipertenso não tratados	7 (4%)	7 (4%)	14	
Creatinina (mg/dL)		1,07	1		0,39
Ureia (mg/dL)		39	40		0,73
HDL (mg/dL)		40	45		0,001
Colesterol total (mg/dL)		166	157		0,22
Triglicérides (mg/dL)		172	122		0,001
Glicemia (mg/dL)		173	93		0,001
Idade (anos)		66	68		0,5
Cianose de membros		25 (16%)	22 (14%)	47	0,09
Edema de membros		17 (11%)	7 (4%)	24	0,07
Rutherford	3	9 (5%)	15 (9%)	24 (15%)	0,244
	4	20 (13%)	18 (11%)	38 (26%)	
	5	41 (26%)	25 (16%)	66 (43%)	0,018
	6	19 (12%)	6 (3%)	25 (16%)	
	Total	89	64	153	0,038
Gangrena de membros		10 (6%)	1 (0,6%)	11	0,01
Infecção de membros		32 (20%)	14 (9%)	46	0,02
Úlcera isquêmica		48 (31%)	28 (18%)	76	0,06
Úlcera mista		14 (9%)	5 (3%)	19	0,07
Dor em repouso		37 (24%)	34(22%)	71	0,40
Angina estável		2 (1%)	0	2	0,22
IAM prévio		18 (11%)	6 (3%)	24	0,03
AVC prévio		12 (7%)	2 (1%)	14	0,02
Total		89 (58%)	64 (41%)	153	

AVC: acidente vascular cerebral; IAM: infarto agudo do miocárdio. * = p < 0,05.

No setor aorto-ilíaco (Figura 3), o escore de Bollinger et al. mostrou médias de pontuações totais (estenoses + oclusões) significativamente maiores nos não diabéticos apenas na artéria ilíaca interna (pontuação média de 4,4 vs. 2,9 pontos; p = 0,001). Na avaliação TASC II do setor aorto-ilíaco, por sua vez, não houve diferenças significativas entre os dois grupos (p = 0,051), embora tenha chegado perto da significância.

**Figura 3:** Comparação dos resultados do setor aorto-ilíaco entre diabéticos e não diabéticos pelos escores TASCII e de Bollinger et al. de acordo com as artérias analisadas. *= p < 0,05.

No setor fêmoro-poplíteo (Figura 4), o escore de Bollinger et al. apresentou maior pontuação total na artéria femoral profunda (pontuação média de 3,7 vs. 4,0; p = 0,04) e na artéria femoral superficial (pontuação média de 8,3 vs. 10,9; p = 0,008) nos não diabéticos. Já o escore TASC II apresentou maior frequência de classificações graves infrapatelares (TASC II C e D) entre os não diabéticos do que entre os diabéticos (p = 0,019).

**Figura 4:** Comparação dos resultados do setor fêmoro-poplíteo entre diabéticos e não diabéticos pelos escores TASCII e de Bollinger et al. de acordo com as artérias analisadas. *= p < 0,05.

No setor infrapatelar (Figura 5), o escore de Bollinger et al. mostrou médias de estenoses > 50% na artéria tibial anterior (pontuação média de 0,7 vs. 0,4; p = 0,005) e de 25-50% nas artérias fibulares (pontuação média de 0,8 vs. 0,5; p = 0,04) e tibiais posteriores (pontuação média de 0,5 vs. 0,3; p = 0,04) nos pacientes diabéticos, mas sem diferença significativa na pontuação total dessas artérias. A Figura 5 mostra os escores totais de cada artéria, somando as estenoses e as oclusões.

**Figura 5:** Resultados dos escores de Bollinger et al. e Bollinger et al. modificado no setor infrapatelar entre diabéticos e não diabéticos de acordo com as artérias analisadas. *= p < 0,05.

No escore de Bollinger et al. modificado (Figura 5), o único dado estaticamente significativo foi o acometimento poplíteo, que se mostrou mais acentuado em pacientes diabéticos (pontuação média de 5,0 vs. 3,3; p = 0,02). A artéria tibial anterior apresentou maiores pontuações nos diabéticos, embora sem significância estatística (p = 0,06).

## DISCUSSÃO

Dentro do objetivo do presente estudo, observou-se, em geral, acometimento arterial distal mais frequente nos diabéticos e lesões proximais mais frequentemente nos não diabéticos. Entre os principais fatores de risco, o tabagismo foi menos frequente nos diabéticos, as dislipidemias foram mais frequentes nos diabéticos e a HAS foi similar nos dois grupos.

Embora não fosse objetivo do estudo, houve predominância significativa da etnia branca em ambos os grupos^30^^-^ 32. A proporção de determinado tipo e etnia e sua relação com doenças cardiovasculares varia muito conforme o continente e a região^32^^,^ 33.

O tabagismo foi menos frequente nos pacientes diabéticos, porém a carga tabágica dos tabagistas e ex-tabagistas foi similar. Esses dados mostram que o tabagismo por si só foi um fator de risco importante para o desenvolvimento de DAP em todos os pacientes. A associação de tabagismo com DM, HAS e dislipidemia é cumulativa no desenvolvimento da doença vascular^9^. O presente estudo vai ao encontro da literatura em relação aos dados demográficos. Entretanto, Santos et al.^18^ encontraram um número maior de tabagistas no grupo dos não diabéticos, grupo que exige maior atenção às mudanças no estilo de vida e por ser o tabagismo um fator de risco independente para o desenvolvimento de DAP.

A dislipidemia foi mais frequente nos diabéticos (colesterol HDL e triglicérides). Entretanto, todos os pacientes do serviço com DAP recebem prescrição de estatinas, independentemente de terem algum tipo de dislipidemia, o que pode causar interferência nesse tipo de análise. Também não pôde ser verificado no presente estudo o grau de aderência dos pacientes ao medicamento. Nos diabéticos, a dislipidemia, em geral, costuma ser caracterizada por aumento dos triglicérides e diminuição do colesterol HDL, sendo que as concentrações de colesterol LDL geralmente não diferem entre diabéticos e não diabéticos^30^. He et al.^31^, ao estudarem a morfologia das placas ateroscleróticas de diabéticos e não diabéticos, também encontraram valores significativamente maiores de triglicérides e, obviamente, de glicemia em diabéticos. Ao contrário do presente estudo, porém, os valores de colesterol total também estavam aumentados nos diabéticos, mas com valores de HDL semelhantes. Todos esses dados vão de encontro ao caráter de acometimento sistêmico do DM, causando um desbalanceamento do perfil lipídico mais evidente no valor de triglicérides^32^.

As infeções e gangrenas úmidas costumam ser mais frequentes nos pacientes diabéticos^16^^,^ 33, em especial no nosso meio, devido a dificuldades de acesso, baixo nível socioeconômico e falta de informação^34^, fazendo com que esses pacientes sejam mais susceptíveis ao aparecimento de tais lesões^16^. Os dados dessas alterações no presente estudo foram apenas referenciais, pois o foco eram as lesões arteriais.

Assim, no TASC II aorto-ilíaco, não foram verificadas diferenças significativas entre os dois grupos. Esse setor compreende artérias mais calibrosas e, talvez, os depósitos de cálcio, mais comuns entre diabéticos, tenham promovido maior perviedade local. Como essa análise não foi significativa, pode ser que realmente não haja diferença entre os grupos ou que a amostra não tenha sido suficiente para tal. O escore de Bollinger et al. para esse setor foi, no geral, similar ao TASC II, mas um pouco mais sensível na artéria ilíaca interna, com pontuação maior (significativa) nos não diabéticos. Não vislumbramos alguma explicação para essa particularidade. Poucos estudos prévios avaliaram comparativamente esse setor entre diabéticos e não diabéticos, pois a incidência de lesões nesses locais acaba sendo baixa, o que dificulta avaliações estatísticas precisas.

Estudos prévios mostraram acometimentos arteriais mais graves no setor fêmoro-poplíteo do que no aorto-ilíaco em ambos os grupos^18^^,^ 19 ^,^ 23 ^,^ 24 ^,^ 35, com maior frequência de oclusões do que estenoses. Essa característica foi observada nos estudos de Graziani et al.^23^ (que só avaliaram diabéticos) e Bradbury et al.^35^ (que só avaliaram não diabéticos), sendo que ambos os estudos mostraram maior frequência de estenoses graves e oclusões nesse setor, porém com frequência ainda maior no setor infrapatelar. Já em estudo angiográfico comparativo entre os dois grupos^18^, foi observado que, em amostra de 117 pacientes (87 diabéticos e 74 não diabéticos) com oclusão fêmoro-poplítea, não houve diferença entre diabéticos e não diabéticos em relação ao reenchimento da artéria poplítea. No presente estudo, o escore TASC II no setor fêmoro-poplíteo mostrou maior frequência de classificações graves (TASC II C e D) entre os não diabéticos do que entre os diabéticos, o que fortalece, novamente, a afirmação de que os não diabéticos apresentam um acometimento proximal mais acentuado do que o distal, ao contrário do encontrado em diabéticos. No escore de Bollinger et al., também houve predomínio de lesões nas artérias femoral profunda, femoral superficial e poplíteas entre os não diabéticos. O estreitamento arterial no canal dos adutores e o encapsulamento arterial dentro desse canal musculoaponeurótico são apontados como fatores de favorecimento de placas nesse local^36^. No entanto, não há explicação para o fato de os diabéticos terem comportamento diferente nesse setor.

Estudos prévios 18 ^,^ 19 ^,^ 23 ^,^ 24 ^,^ 35 também mostraram maior frequência de lesões nos pacientes diabéticos no setor infrapatelar. O presente estudo corroborou esses achados com frequência significativamente maior de estenoses > 50% na artéria tibial anterior e estenoses de 25-50% nas artérias fibulares e tibiais posteriores. Entretanto, a pontuação total (estenoses + oclusões) foi similar entre os grupos (Figura 4). Com relação ao acometimento distal, Santos et al.^18^ encontraram frequência similar de opacificação das artérias poplíteas, fibular e tibial anterior entre diabéticos e não diabéticos, sendo que a artéria tibial posterior teve maior frequência de oclusão nos diabéticos (p = 0,008). Em contrapartida, o escore de Bollinger et al. modificado mostrou maior acometimento poplíteo nos diabéticos. Além disso, no estudo de Santos et al.^18^, os diabéticos eram predominantemente do sexo feminino, hipertensos e não fumantes, e a regressão logística mostrou que somente o sexo feminino foi fator de risco para não opacificação da artéria tibial posterior. Jude et al.^24^, em estudo análogo, mostraram que, nos diabéticos, as lesões arteriais foram mais proeminentes na artéria femoral profunda e em todas as infrapatelares, porém os autores não estratificaram qualitativamente as lesões encontradas. Assim, no presente estudo, foi significativa a presença de estenoses nas três principais artérias da perna, porém com associação significativa de estenoses > 50% somente na artéria tibial anterior. As alterações múltiplas em artérias distais podem limitar o sucesso de reconstruções vasculares por deficiência de deságue^37^.

Algumas limitações devem ser assinaladas no presente estudo. Trata-se de um estudo retrospectivo e, como tal, o levantamento de prontuários costuma ter um contingente expressivo de dados incompletos, o que acarreta grande número de exclusões e probabilidade de vieses, enfraquecendo, assim, a qualidade da amostra e as conclusões. Outra limitação foi a impossibilidade de analisar as lesões das extremidades pelas classificações de Wagner, WIFi ou Glass^38^ devido ao caráter retrospectivo do estudo. Embora tenham sido usados escores e checagem por especialistas, um certo grau de subjetividade pode ter ocorrido na avaliação das arteriografias. Os dados de dislipidemias podem ter um viés por conta do uso (em geral irregular) de estatinas pelos pacientes. Os dados não significativos podem ter ocorrido pela ausência real de significância ou porque a amostra foi insuficiente devido ao excesso de perdas. Uma amostra multicêntrica de grandes proporções poderia esclarecer essa controvérsia.

Estudos prospectivos randomizados, em especial multicêntricos, são necessários para melhorar a evidência sobre o perfil diferenciado de acometimento arterial periférico nos diabéticos.

## CONCLUSÃO

Na casuística do presente estudo, o escore de Bollinger et al. mostrou maior pontuação de lesões arteriais nas artérias ilíaca interna, femoral profunda e femoral superficial entre os não diabéticos. Porém, a artéria poplítea teve maior pontuação entre os diabéticos. A classificação TASC II mostrou frequência de classificações no setor aorto-ilíaco similar entre diabéticos e não diabéticos e maior frequência de classificações C e D no setor fêmoro-poplíteo entre não diabéticos.

No segmento infrapatelar, o escore de Bollinger et al. mostrou que pontuações para estenoses foram significativamente maiores nos diabéticos em todas as três artérias distais (tibial anterior, tibial posterior e fibular), mas sem diferença na pontuação total (oclusões + estenoses). O escore de Bollinger et al. modificado mostrou apenas diferença de pontuação na artéria poplítea, sendo a mais acometida nos diabéticos.
